# Genetic Parameters of Somatic Cell Score in Florida Goats Using Single and Multiple Traits Models

**DOI:** 10.3390/ani12081009

**Published:** 2022-04-13

**Authors:** Rocío Jimenez-Granado, Antonio Molina, Chiraz Ziadi, Manuel Sanchez, Eva Muñoz-Mejías, Sebastián Demyda-Peyrás, Alberto Menendez-Buxadera

**Affiliations:** 1 Department of Animal Production, University of Córdoba, 14071 Córdoba, Spain; rociojimenezvet@gmail.com (R.J.-G.); pa1sarom@uco.es (M.S.); 2 Department of Genetics, University of Córdoba, 14071 Córdoba, Spain; ge1moala@uco.es (A.M.); ziadichiraz@yahoo.fr (C.Z.); ambuxadera@yahoo.com (A.M.-B.); 3 Department of Animal Pathology, Animal Production, Bromatology and Food Technology, University of Las Palmas de Gran Canaria, 35001 Las Palmas, Spain; evammejias@gmail.com; 4 Department of Animal Production, National University of La Plata, La Plata 1900, Argentina; 5 National Council of Scientific and Technical Research (CONICET), La Plata 1900, Argentina

**Keywords:** milk, somatic cell count, genetic parameters, mastitis

## Abstract

**Simple Summary:**

Over the past few years, the Florida breed goat improvement program has led to significant genetic progress in milk production. However, there has been a parallel increase in the incidence of mastitis cases in this population. In this study, we try to assess the current situation of the population and estimate the genetic parameters of somatic cell score (SCS) using the dairy test day and single and multiple traits models. Genetic analysis of this score may allow us to select goats that are more resistant to mastitis. The results show that the SCSs have a heritability of medium-high magnitude, which ensures genetic progress if this criterion is used for selection. Finally, from the point of view of selection, the SCS should not be interpreted as an expression of the same trait over all the parturitions, even though they are closely correlated.

**Abstract:**

A total of 1,031,143 records of daily dairy control test of Spanish Florida goats were used for this study. The database was edited, and only the records of the first three lactations were kept. The final database contained 340,654 daily-test somatic cell counts from 27,749 daughters of 941 males and 16,243 goats. The evolution of this count in the last 14 years was analyzed following French and American international associations’ criteria for the risk of mastitis in goats, and confirmed the slight increase in SCS in the last years and the importance of this problem (50% of dairy control tests show a risk of suffering mastitis). For the genetic analysis, the SCS records were log-transformed to normalize this variable. Two strategies were used for the genetic analysis: a univariate animal model for the SCS assuming that SCS does not vary throughout the parities, and a multi-character animal model, where SCS is not considered as the same character in the different parities. The heritabilities (h^2^) were higher in the multiple traits models, showings an upward trend from the first to the third parity (h^2^ between 0.245 to 0.365). The genetic correlations of the same trait, as well as between breeding values (GVs) between different parities, were different from unity. The breeding values (EBVs) obtained for both models were subjected to a PCA: the first eigenvector ($\lambda_{1})$ explained most of the variations (between 74% to 90%), while the second $\lambda_{2}\;$accounted for between 9% to 20% of the variance, which shows that the selection will be proportionally favorable but not equivalent in all parities and that there are some variations in the type of response.

## 1. Introduction

Florida is a native Spanish goat breed distributed mostly in central and southern Spain, although there are herds in other countries such as France, Italy, or Portugal. This breed is raised under a wide variety of production systems, ranging from semi-extensive to intensive systems. The breed has a census of about 125,000 breeding goats, of which 30,000 are registered in the national herdbook [1].

Nowadays, the genetic improvement program for the Florida breed is based mainly on milk production and milk composition traits [2,3,4] or morphological and reproductive traits [5,6]. In these traits, important genetic progress has been made, especially in milk production (expressed as cumulate milk production at 240 days of lactation), with an annual increase of 1.7% over the last 10 years. However, the selection of goats to increase their level of milk production leads to a greater, more constant interaction between the udder and the milking equipment, thus increasing the likelihood of mammary gland infections, together with greater sensitivity of the goats to infectious agents, due to the productive stress they suffer. As in the case of dairy cattle, we might suspect that the incidence of mastitis is increased in goats [7].

Since there is no clinical evidence in most cases of mastitis (subclinical mastitis), it is difficult to diagnose, which makes treatment and prevention all the more difficult. Nevertheless, the somatic cell count (SCC) has been extensively used as an indirect indicator of mastitis. The somatic cells in milk are leukocytes (neutrophils, eosinophils, macrophages, lymphocytes) derived from blood circulation, as well as cellular debris and mammary epithelial cells, the former being the most common situation in ruminants [8].

SCC levels can be affected by multiple factors, and these data do not allow us to make a clear distinction between chronic or repetitive prevalence [9]. In addition, in the case of sheep and goats, there are not enough studies to determine a clear relationship between SCC and the probability of suffering mastitis [10,11]. According to [12], SCC monitoring and analysis should be interpreted as a sign of a major multifactorial problem of inflammation of the mammary gland, since it is not possible to distinguish the threshold level of subclinical mastitis or to tell which quarter of the udder is inflamed.

Although progress has been made in preventive measures for its control, high levels of SCC may have negative effects on milk processing, which leads to significant economic losses both at the industry level and in the herd itself [13]. The objective of an abnormal milk control program is to prevent abnormal milk from entering the channels destined for human consumption. The milk somatic cell count (MSCC) is the basis for abnormal milk control programs in cows, goats, and sheep. In the United States of America (USA), the legal MSCC limit established by the Food and Drug Administration for cows is 750,000 mL^−1^ and for goats and sheep, 1,000,000 mL^−1^. In the European Union (EU) (EC Regulation No. 853/2004) [14] the legal limit for cows is 400,000 mL^−1^, and there is no legal limit for goats and sheep.

The somatic cell count is a phenotypic expression of the presence or absence of a mammary infection and there is solid evidence that this trait can be a key tool for indirect selection for greater resistance to mastitis [15]. However, this type of infection is multiple and can manifest itself at different levels [16], and therefore the databases must contain detailed information, which is not always available, particularly in goats. The few published references for this species express the results in terms of averages per lactation [17] and recently, according to the periodic records applied in breeding programs [18]. Although there is some coherence in the results of these estimates, a comparison between them is urgently needed.

On a practical level, two protocols have been established in the case of dairy goats to assess the current situation of herds based on the somatic cell count. According to Renée de Crémoux [19], in studies carried out at the French Breeding Institute (Institut de l’Elévage), a goat is considered healthy when the SCC throughout lactation is less than 750,000 cells/mL, with values over 2 million somatic cells indicating clinical mastitis and intermediate values showing subclinical mastitis. The second protocol is applied in USA dairy goats [20], by which goats with over 1 million somatic cells/mL would have a high probability of suffering mastitis. 

The objectives of this study were therefore to determine the incidence of clinical and subclinical mastitis in the Florida goat population (prototype of the Spanish dairy goat breed), by analyzing the somatic cell count (an indirect indicator of the incidence of clinical and subclinical mastitis); and to estimate the effects of environmental variation and the genetic (co)variance components of the somatic cell count during the first three parities in this breed, comparing those estimations using single and multiple traits animal model models.

## 2. Materials and Methods

### 2.1. Phenotypic Data

For this study, a total of 1,031,143 test day (TD) records collected between 2005 and 2019 were accessed from the National Association of Florida Goat Breeders (ACRIFLOR). These TD records belonged to 90 herds and included a total of 58,606 females. All the data were subjected to a data-editing process, and all TDs longer than 305 days, parity numbers greater than 6, and records with Somatic Cell Count (SCC) outside the usual range (<40 × 10^3^ to >10,000 × 10^3^) were excluded. 

Next, SCC was transformed by SCS = log_2_(SCC) + 3 [21], and any somatic cell score (SCS) records outside the range of ±3 standard deviation were removed. The SCS variable was studied according to the results of each test day control (SCSTD) and as its geometrical average for each of the lactations (SCSPT). The resulting data from this process was associated with the pedigree available from ACRIFLOR (109,583 goats), leaving a total of 805,373 observations making up the TOTAL database. This was used to perform a preliminary analysis of the evolution of estimated clinical and subclinical mastitis according to the French [19] and American [20] criteria, through the analysis of the somatic cell count.

The data sets for the genetic analysis (PROD set) were created from the TOTAL database, and only the information from the first three lactations was selected, with the goats with less than 3 TD in each of the first three parities, the combinations of herds with test dates (gfc) with less than 10 TD and parents with less than 10 progenies being excluded. After this sequential process, 340,654 TD of the first three parities from 90 herds of 27,479 daughters of 941 sires and 16,243 dams were available, of which 8788 were in the data vector (Table 1). The results of the somatic cell count were studied using two approaches: comparing them with the results of each TD (file PROD1, variable SCSTD) and the geometrical average of each lactation (file PROD2, variable SCSPT).

### 2.2. Statistical Analysis

Records of the dependent variable of the total file were analyzed, using a fixed-effects linear model to obtain initial information on the causes of variation that affect the SCS results. In this first analysis, we considered the fixed effects of herd, year and month of test, litter size (ls_j_ j = 4 classes: single, double, triple, and four or more), and parity number (pn_k_ k = 1 to 6 or more parities).

The Asreml 3 software were used to estimate the genetic (co)variance components. The PROD1 and PROD2 files and two possible scenarios were compared, based on the considerations and form of expression of the SCS original variable:

- Univariate approach. The parameters for SCSTD and SCSPT were estimated with the total available data (PROD1) using a single linear model (UVM), assuming that they did not vary throughout parities:
  Model 1: SCS evolution through lactation using SCSTD in each lactation
  Model 2: Evolution of the average SCS per lactation using SCSPT throughout the first three lactations.
- Multivariate approach. The parameters for SCSTD and SCSPT were estimated with the total data available from each parity (PROD2), using a multiple trait linear model (MTM), assuming that they were not the same trait across parities.
  Model 3: Evolution of SCS through lactation using SCSTD (with each SCS TD as a different trait)
  Model 4: Evolution of the average SCS per lactation throughout the different lactations (1 to 3), using SCSPT and considering them as different traits.

In statistical terms, the general representation was very similar:

(1) $$y_{i}=\mathrm{Xb}+Z_{1}a+Z_{2}p+e$$

where y_i_ is a vector of the dependent variables SCSTD and SCSPT; b is a vector of previously defined fixed effects (gfc_i_, with 4133 and 4472 levels for SCSTD and SCSPT, respectively); ls_j_ and pn_k_)), although the levels of i and j were not equal. a, p, and e are vectors of random effects due to the goat genetic effects with data and their ancestors without records (a with 40,604 levels); permanent environment due to the repeated measurements in the goat (p with 27,749 levels) and residual error (e with the number of levels depending on the model), respectively.

The matrices X, Z_1_, and Z_2_ are incidence matrices that relate the fixed and random effects with the dependent variables. The expected variances are:

(2) $$\mathrm{var}\left[\begin{array}{c} a\\ w\\ e \end{array}\right]=\left[\begin{array}{ccc} A⊗G_{a} & 0 & 0\\ 0 & I⊗W_{p} & 0\\ 0 & 0 & e \end{array}\right]$$

in which A is the numerator of the relationship matrix between the goats with data and their ancestors without data; G_a_ and W_p_ are matrices of (co)variance of additive genetic and permanent environmental effects; e is the residual effects matrix. I is an incidence matrix with an order equal to the p^th^ number of goats with data and ⊗ is a Kronecker operator. To facilitate the estimates, it was assumed that the dependent variable studied in each lactation corresponds to repeated measures of the same traits, and using the facilities of ASReml 3 with the option of longitudinal models, the same components of (co)variance are estimated as a classic MTM model saving computing time. It is necessary to take this approach into account to understand the inclusion of the permanent environment effect in the MTM models.

The elements of the matrices Ga and e depend on this approach and are represented as follows: in the case of UVM: G_a_ = $\sigma_{a}^{2}$ and e = $\sigma_{e}^{2}$, whereas, for MTM,

(3) $$\mathrm{Ga}=\left[\begin{array}{ccc} \sigma_{a_{k_{1}}}^{2} & \sigma_{a_{k_{12}}} & \sigma_{a_{k_{13}}}\\ \sigma_{a_{k_{21}}} & \sigma_{a_{k_{2}}}^{2} & \sigma_{a_{k_{23}}}\\ \sigma_{a_{k_{31}}} & \sigma_{a_{k_{32}}}. & \sigma_{a_{k_{3}}}^{2} \end{array}\right]\;\mathrm{and}\;e=\left[\begin{array}{c} \sigma_{e_{k_{1}}}^{2}\\ \sigma_{e_{k_{2}}}^{2}\\ \sigma_{e_{k_{3}}}^{2} \end{array}\right].$$

where $\sigma_{a}^{2}$ and $\sigma_{e}^{2}$ are the additive genetic and the residual variances, respectively, and $\sigma_{a_{k_{i}}}$ are the (co)variances between k_i_ parities.

The heritability values (h^2^) will be estimated by the classic formulas:

(4) $$h^{2}=\frac{\sigma_{a}^{2}}{\sigma_{a}^{2}+\sigma_{p}^{2}+\sigma_{e}^{2}}\;$$

and

(5) $$\;h^{2}=\frac{\sigma_{a_{k_{i}}}^{2}}{\sigma_{a_{k_{i}}}^{2}+\sigma_{p}^{2}+\sigma_{e_{k_{i}}}^{2}}$$

for the total univariate and multivariate approach, respectively: it should be noted that the residual variance is not the same, while the genetic correlation (r_g_) between the same trait in the different parities will be:

(6) $$r_{g}=\frac{\sigma_{a_{k_{\mathrm{ij}}}}}{\sqrt{\sigma_{a_{k_{i}}}^{2}{*\sigma }_{a_{k_{j}}}^{2}}}$$

The repeatability estimates (Rep) will be estimated in a similar way to h^2^, except that the numerator includes the permanent environmental variance ($\sigma_{p}^{2}$), which was defined as a cumulative effect following the results from Schaeffer [22]. 

The large number of EBV results for SCSTD and SCSPT in each parity can be combined in a selection index; however, the economic importance of each expression of SCS is not known, to obtain a solution the procedure presented by Togashi and Lin [23] was used. To do that the results of each EBV were subjected to a principal component analysis [24] to identify a new underlying variable that would explain most of the variances of each variable (SCSTD and SCSPT), which can be identified in the eigenvalues. The corresponding eigenvectors coefficients (ev_i_) can be used as a weighting factor to estimate a new underlying index that can serve as a guide for the selection of healthier goats in all the parities. This new index, represented by the symbol Ipc, is estimated as follows:

(7) $$I_{\mathrm{PCT}}={\mathrm{ev}}_{1}^{\prime }{*\mathrm{BV}}_{1}+{\mathrm{ev}}_{2}^{\prime }{*\mathrm{BV}}_{2}+{\mathrm{ev}}_{3}^{\prime }{*\mathrm{BV}}_{3}$$

where ev_1_, ev_2_, and ev_3_ are the coefficients of the ev_i_ and the EBVi are the genetic values of each goat for each parity expressed in a standardized form. The correlations between the original variables and the I_PC_ will be estimated as follows:

$$r_{\mathrm{BV},I_{\mathrm{PC}}}={\mathrm{ev}}_{i}\sqrt{\lambda_{i}}$$

where $\sqrt{\lambda_{i}}$ is the square root of the corresponding eigenvalue.

## 3. Results and Discussion

It is widely accepted that mammary inflammation, generally of an infectious origin, is the main factor in increasing the somatic cell count (SCC) [25], besides causing negative effects on milk production and quality in small ruminants [26,27]. For this reason, SCC is commonly used in cattle as a sensitive marker of udder health conditions and as a commercial milk quality parameter in bulk tank milk. However, to be able to interpret SCC properly in small ruminants, it is also necessary to take into account the influence of other factors apart from infections [28,29]. In this work, we analyzed the main environmental and genetic effects that determine the somatic cell count in milk throughout and between lactations of the Florida goat. 

**Figure 1:**
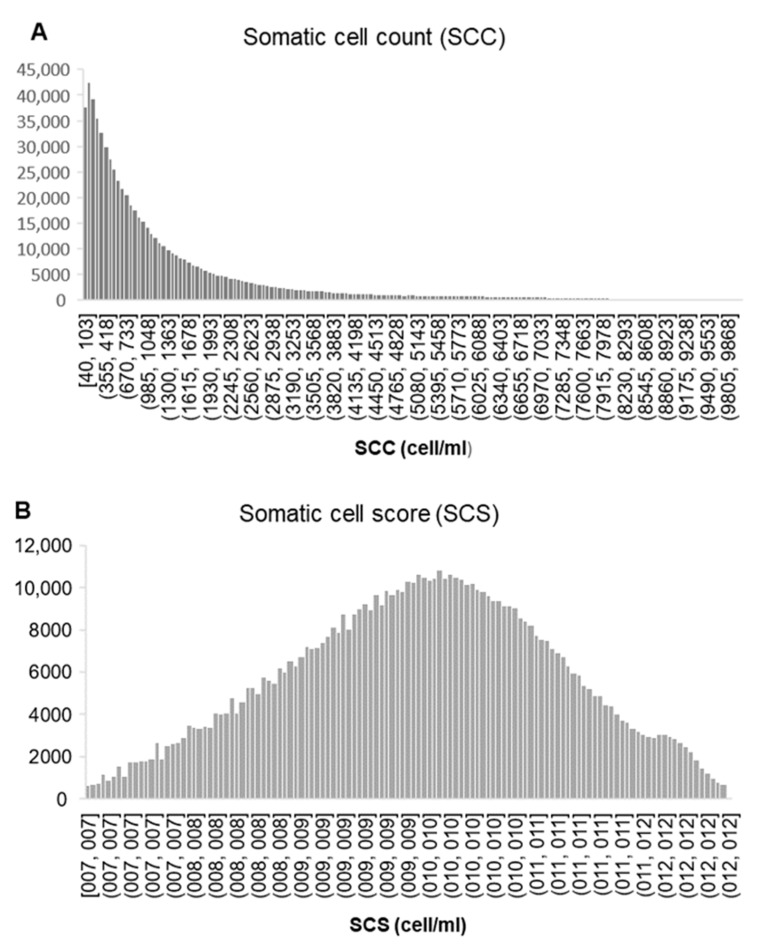
Frequency distribution of the variable somatic cell count (SCC) in Florida goat before (**A**), and after logarithmic transformation (SCS, (**B**)).

Somatic cell count data usually has a non-normal distribution (a skewed left distribution), as can be observed in Figure 1a. Next, we applied a logarithmic transformation, as suggested by earlier studies, and as can be observed, normal distribution was achieved (Figure 1b).

### 3.1. Estimating the Incidence Level of Mastitis

Although in dairy cattle, somatic cells score (SCS) is a milk quality indicator widely used to predict subclinical mastitis levels, few studies currently exist that confirm this association in the case of goats. However, there are currently several study protocols that confirm the correlation between cell count and the presence of mastitis (clinical or subclinical). Such is the case of the French goat [19], which has two clearly-defined thresholds:

- 750,000 SC/mL: below this count, a goat is considered healthy
- 750,000–2 million SC/mL: moderate inflammation; subclinical mastitis
- 2 million SC/mL: above this count, a goat has severe inflammation; clinical mastitis.

In USA dairy goats [20], the permitted level for goat milk is 1 million SC/mL, with a count level over one million/mL indicating the presence of mastitis. This threshold is significantly higher than that used in dairy cattle (280,000 SC/ mL).

**Figure 2:**
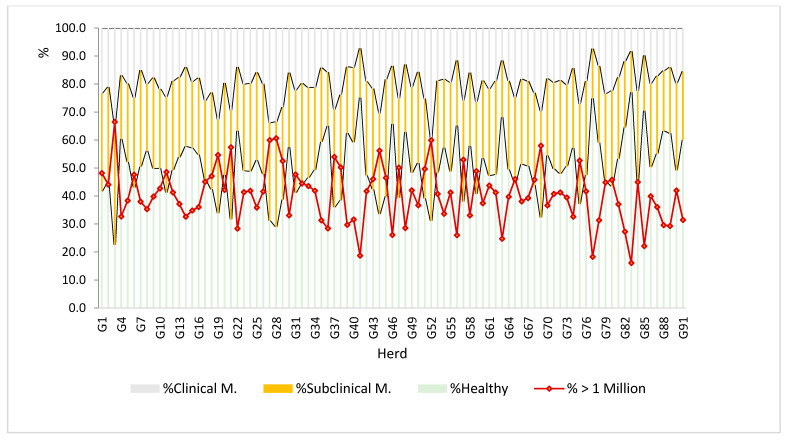
Estimation of presence (subclinical and clinical) or absence of mastitis, based on the Paape et al. 2001 criteria (healthy/>1 million suspected of mastitis) and Renée de Crémoux, 2012 (healthy, subclinical, or clinical mastitis) in each of the Florida breed farms in the study.

The global analysis by farm using the previous thresholds in the Florida breed (Figure 2) allows us to verify the existing problem in this breed (in the Spanish goat at least).

Thus, following the criteria of Renée de Cremoux [19], it can be observed that about 50% of the control tests carried out in these herds present an average number of cells higher than the threshold of 750,000 cells/mL per test, with 40% of the herds presenting average levels above this threshold. Following this standard, the average percentage of tests exceeding the threshold for subclinical mastitis was 30% (72% of the herds presented a level higher than this threshold), and 20% for clinical mastitis (16% of herds presented averages above the threshold for clinical mastitis). The seriousness of this fact lies not only in the obvious losses due to mastitis (decrease in milk production, need for extra treatments, and quarter dry-off among others) but also from less evident economic losses due to the much more frequent subclinical mastitis. In this way, the quality of the products made from said milk could be affected. After performing the analysis following the criteria of Paape et al. [20], which establishes a healthy/suspicious threshold of one million cells/mL for each control test, the average percentage of tests that exceeded this level was over 40%, with the averages of 14 herds (of the 90 analyzed) exceeding this level.

**Figure 3:**
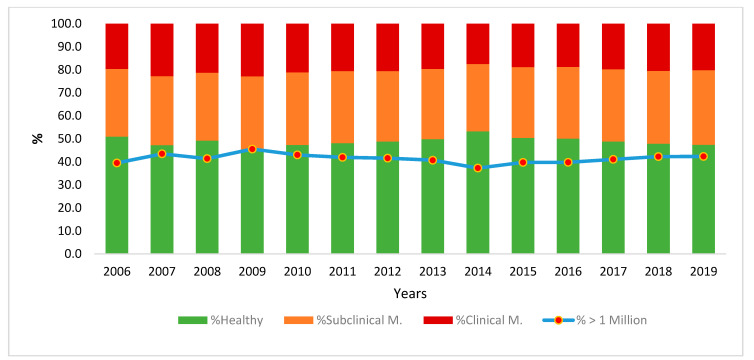
Estimation of the evolution of subclinical and clinical mastitis throughout the study period (or absence of mastitis) based on criteria of Paape et al., 2001 (healthy/>1 million suspected of mastitis) and Renée de Crémoux, 2012 (healthy, subclinical, or clinical mastitis).

The evolution of these parameters in the period analyzed (14 years) allows us to confirm the gravity of the problem (Figure 3): far from being a specific problem of one particular period, it can be considered a systemic problem of the goat, with control test levels above the threshold of 750,000 cells in over 50% of the tests (with annual averages of 46 to 55% of the tests for one year). The evolution over the last five years seems to show that the problem is becoming worse, with the levels exceeding the threshold in 52% of the control tests over the last two years. This fact coincides with a simultaneous increase in the percentage of estimated cases of mastitis, with averages above two million cells per mL in 20% of the tests. Finally, taking into account the criteria of Paape et al. [20], 41% of the tests showed values above the threshold of one million cells (in all the years analyzed, more than 25% of the test values showed an SCC above the threshold of one million cells).

**Figure 4:**
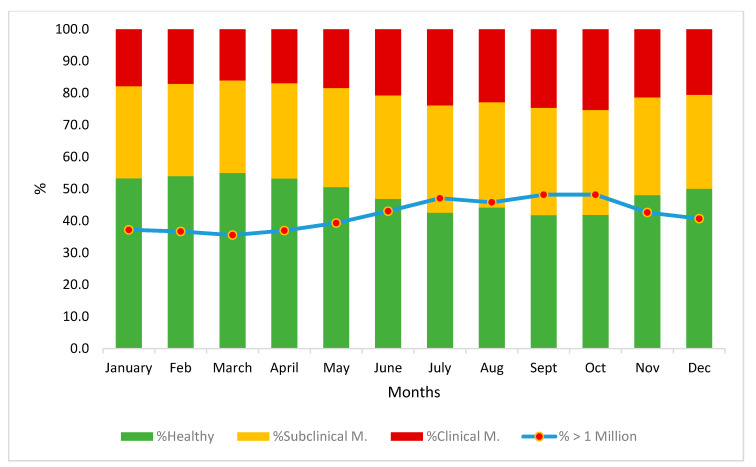
Estimation of the evolution of subclinical and clinical mastitis throughout the year (presence or absence of mastitis) based on criteria of Paape et al., 2001 (healthy > 1 million as suspected of mastitis) and Renée de Crémoux, 2012 (healthy, subclinical, or clinical mastitis).

Figure 4 shows the average evolution throughout the lactation period. The average SCC in control tests below 750,000 cells ranges from 41% (in September and October, the months with the highest incidence of mastitis) to 55% in March (the month with the lowest incidence). The incidence of clinical mastitis was higher between June to December, with over 20% of the control tests above 2 million cells, compared to the first 6 months of the year, which showed an incidence of under 20%, while the incidence of subclinical mastitis exceeded 30% in the tests from July to October, with just under 45% of the tests surpassing the 1 million cell mark. Finally, the months of June to December presented the highest levels of suspected mastitis in the tests according to the criteria of Paape et al. [20], with over 40% of the tests above the level of one million cells/mL. Of these, the months of September and October were the worst, with almost 50% of the tests above the one million cell level.

### 3.2. Preliminary Analysis of Environmental Effects on SCS

In general terms, the level of susceptibility to mastitis in the Florida breed (Table 1) is within the range published in other European countries [9]. The results of the fixed effects model with the TOTAL file were highly significant for all the causes of variation included (results not shown). However, there is high variability between farms (*p* < 0.001), reflecting different sanitary conditions and the handling of the goats, especially of the milking equipment and routines, which are not homogeneous in all the herds, as occurs in other Spanish breeds.

In the same way, there was a considerable difference in the average SCSs over the years (*p* = 0.001). The evolution of this variable throughout the analyzed period showed an increase until 2009, with a fall up to 2014, and an approximately linear increase up to the present day, except for last year.

Among the possible factors affecting this evolution (especially the decrease from 2009 to 2014) is the sharp decrease in the price of milk, which led to a decrease in farm censuses, with a large number of older goats with higher levels of SCS being culled, and fewer replacements; the younger goats, therefore, had lower levels of SCS.

Once the population had stabilized, there was a clear increase, which shows the high incidence of mastitis that exists today. In the same way, it shows the existence of a very marked seasonal pattern in SCS levels, with maximum values in summer and minimum values in winter. Jimenez-Granado et al. [29] demonstrated in this same breed that one of the environmental effects that most affects SCS is the month of parity, so the control tests that take place in the summer months produce a rise in SCS compared with parities during the rest of the year. Responsibility for this result is attached by these authors to the higher temperatures during these months and the lower milk production in summer. This same pattern was presented by [30] in the Serrana breed in Portugal and in the Payoya breed in Spain [31], which these authors also ascribed to climatic effects. Other authors indicate that this type of response may be due to other effects, such as the photoperiod [32], which produces stress in the lactating goat, which also affects its productive levels [28]. In this way, in the spring (with an increasing photoperiod, mild temperatures, and sometimes better feeding), the goats’ milk production tends to increase, and the SCC decreases (expressed by somatic cells per mL of milk). More details on the causes of somatic cell count variation in dairy goats can be found in Jimenez-Granado et al. [33]. The least-squares means evolution analysis for the parity number showed the level of SCS increases in line with the number of parities with goats with four parities or more, with a rise of +0.801 in the SCS (equivalent to +825,600 somatic cells compared to the first parity).

According to Jimenez-Granado et al. [29], the number of lactations has an important influence on the variation of the SCS, so that older goats (which generally coincide with those with the highest number of lactations) present higher values in the SCS than their younger counterparts. This could be attributed to a parallel increase in subclinical infections and/or trauma caused by milking that favors the infiltration of microorganisms. Similar results were obtained in this same breed by Jimenez-Granado et al. [29] in goats in New Zealand [34,35], Poland [36,37], and the United States [38]. It has been suggested that this response is due to physiological changes in successive lactations leading to a higher level of udder infections [39].

Our results also showed how the SCS level increases with litter size, with a positive difference of 461.55 between multiple litters of four kids and a simple litter. According to Jimenez-Granado et al. [29], in the analysis of SCS according to the type of kidding, it is important to separate the effect of the type of kidding from the lactation number effect. For instance, it is very common for primiparous females to give birth and raise a single kid, presenting lower SCS than multiparous females.

### 3.3. Genetic Analysis of SCS throughout Lactation and between Lactations

The results of the estimates of the (co)variance components for the single and multiple traits models are presented in Table 2 and Table 3, respectively.

In both forms of expression at the level of somatic cells, the multiple models showed results for the (co)variance components, as well as levels of h^2^ and Rep (Table 3) higher than the univariate model (Table 2). Although there are not many references for the h^2^ and Rep values of this trait in goats, the estimated parameters were very similar in general terms to the publications by Scholtens et al. [34,35] in goats exploited under New Zealand conditions, while the estimates of h^2^ for the SCSTD results were within the range presented by Arnal et al. [40] in the Alpine and Saanen breeds in France and by Rupp et al. [41] for SCSPT. 

The parameters presented in Table 3 indicated that the components of (co)variance do not remain constant throughout the deliveries. This trend is corroborated by the results of the principal component (PC) analysis of the matrix of genetic components in the MTM model (lower part of Table 3), which yielded a different structure, particularly in the relative importance of PC2, which was twice as high for SCSTD versus SCSPT. These components of PC2 are generally known as ‘shape vectors’, which explains the differences in the genetic variances of each parity in both forms of expression.

This type of response, as well as the results of the genetic correlations showed in Table 4, has two consequences. On the one hand, the somatic cell count levels expressed as the average of each lactation (SCSPT) bias the results by not taking into account the existence of variations between parities, which is more clearly evident when the results of each periodic control test (SCSTD) are used. On the other hand, these results also imply that, in genetic assessments of resistance to mastitis, it should not be assumed that somatic cell count levels indicate a similar improvement throughout parities. No similar references have been found in goats, although the same trend is found in sheep [42], as well as in dairy cattle [36,43].

**Figure 5:**
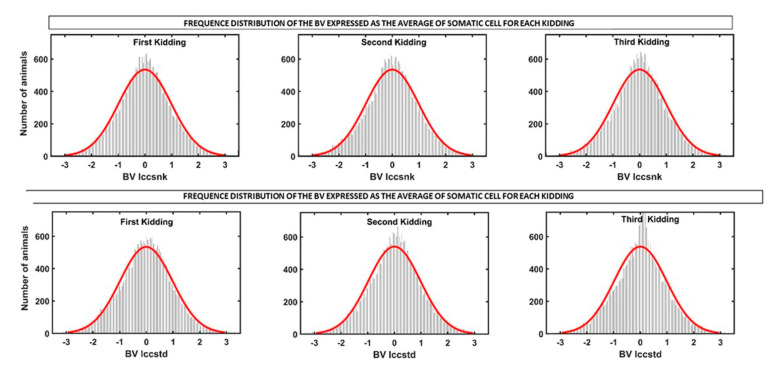
Frequency distribution of the breeding value for SCSTD and SCSPT of all goats throughout the first three parities.

Figure 5 illustrates the existing variability in the EBV frequency distribution of all goats for both variables in each of the first three parities (standardized breeding values). Considering the results shown in Table 1 and Table 3, the genetic coefficient variation was 4.3 to 4.9% for SCSPT and between 6.9 to 7.4% in SCSTD, which are high enough values to be considered useful for the breeding program in this breed.

The results of the principal component analysis (APC) of the EBV indicate that the first λ explains most of the variances of the EBV and the vectors for SCSTD and SCSPT for each parity have positive coefficients and are closely related. These vectors represent the general level of somatic cells in all parities, so that if the selection favors the EBV results in a single parity, the response will be proportionally favorable but not proportional in the rest, with some variations. However, although the vectors for each parity are located in different quadrants, they evolve in the same direction (indicating that both forms of somatic cell count expression should be treated as the same trait within each parity), while the records of each birth should be considered as different (but genetically correlated) characters. This trend can be corroborated if we consider that the vector form (second λ) is responsible for between 9% and 20% of the variance among all EBV, and variations in the response form should therefore be expected. 

**Figure 6:**
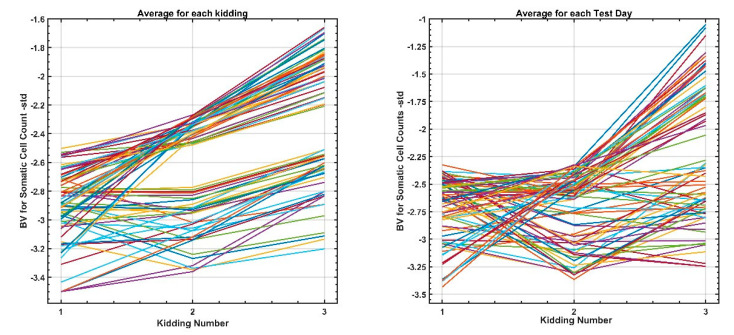
Variations in breeding values for all parities for the 500 best goats selected in the first parity.

To exemplify this trend, the best 500 goats were selected according to their EBV for SCSTD and SCSPT in the first parity (see Figure 5), and the results in their second and third parities were carefully examined (Figure 6).

The results for SCSTD and SCSPT indicated that the response will be positive in all parities. However, within these groups of elite goats, it has been possible to identify goats with stable behavior that maintain the same level in the three parities, while in others, which can be considered “sensitive” to the effect of the number of parities, the genetic level decreases in the second and third parity. No similar references to this approach have been found in the study of somatic cells. However, in biological terms, this type of response, where the capacity of the goats can vary along a time scale or environment has been termed plasticity [44], a term which has been widely applied in the field of goat improvement (reviewed by Jong and Bijma [45]).

**Figure 7:**
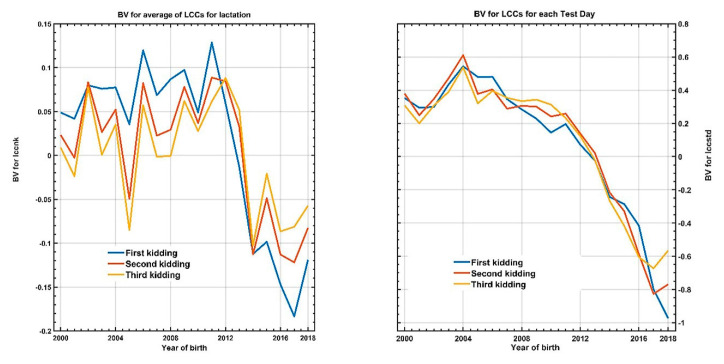
Observed evolution of EBV for both expressions levels of somatic cell count in each parity, as a function of the goats’ year of birth.

During the period represented, the level of somatic cells showed a decrease of −1.39 std (equivalent to -958 SCC counts) in the EBV of this studied population. Figure 7 shows these results for both expressions of somatic cell count and these curves represent the evolution of the EBV (expressed in std) in the breeding program of this breed over almost two decades. Since there is no direct selection for a lower SCC, this decrease is most likely due to an improvement in udder morphology. In fact, for almost a decade, the linear type evaluation has been applied in this breed, which allows the selection by farmers of goats with a better-shaped udder (variables such as the depth of the udder or the suspensory ligament medium are closely correlated with somatic cell count).

The results show the same pattern, with depressive responses (favorable effects) depending on the year of parity, but greater uniformity when the records of each test day are used. For instance, the publications by Rupp et al. [46,47] on divergent selection in sheep and goats, respectively, indicated that the levels of somatic cell counts could be amended through genetic improvement, and the results found in this study are consistent with that view.

Given that, for practical purposes, the use of EBV is not recommended for each parity, we propose a solution by applying a principal component analysis (APC) to the EBV estimates of both expressions of the somatic cell count according to the procedure proposed by Togashi and Lin [23]. This consists of the use of the eigenvector coefficients (ev_i_) of the most important eigenvalues ($\lambda_{i}$) as a weighting factor in the construction of an underlying index. The ev_i_ are orthogonal and uncorrelated, and can therefore be used to prepare the Ipct already presented in the Materials and Methods section, the results of which are shown in Table 5.

The elements in Table 5 bear a close resemblance to the ev_i_, coefficients, while the differences are expressed when the Ipct and correlations are made with the original variables, which show higher values than those previously presented (see Table 3 and Table 4). These differences are because Ipct retains most of the (co)variances existing in both variables throughout the parities. As a result, this alternative can be useful in the improvement program, since a single indicator synthesizes all the information. This approach has been successfully applied in various goat selection scenarios in dairy cattle [48] and in meat production [49].

## 4. Conclusions

The Florida breed goat improvement program has led to significant genetic progress in milk production, but there has been a parallel increase in the incidence of mastitis in this population. The lack of a systematic detection protocol, especially for subclinical mastitis, in this breed avoids genetic evaluation for resistance to this disease. The analysis of the somatic cell count can allow this assessment. Our results show that if the somatic cell count, whether recorded as an average of each lactation or on the test day, has significant genetic variability, it can provide important benefits to the program to improve resistance to mastitis in Florida goats. However, the SCC cannot be interpreted as an expression of the same trait throughout the parities (although they are closely correlated) in the genetic evaluations. The levels of heritabilities and the correlations of this trait in the different parities are consistent with the few available references on this species and follow the same trend as the publications on sheep and dairy cows. Further studies are required to improve the estimates of the genetic (co)variance components through longitudinal models and to consider genetic correlations with other traits of economic interest. In the same way, a more precise definition of the necessary protocols in the testing systems for this type of trait is needed for their subsequent use in the genetic evaluation of resistance to mastitis in dairy goats. In that way, it is also advisable to carry out specific analysis that demonstrate the association of this count with the incidence of subline mastitis in this breed, as has been conducted in French and North American goats. This analysis will allow for the establishment of specific limits for this breed that determine a high probability of suffering from the disease.

## Figures and Tables

**Table 1:** Number of goats and records by parity used in the SCC (somatic cell count) genetic analysis (standard error between parenthesis).

	Parity			Total
	First	Second	Third	
No. of goats	25,430	19,268	12,599	27,749
No. of sires	941	900	807	941
No^.^ of dams	15,215	12,150	8,406	16,243
No. of TD records	145,816	118,039	76,799	340,654
Average SCC (×10^3^)	972.44 (3.02)	1249.45 (3.71)	1483.02 (4.69)	1193.96 (2.14)

**Table 2:** Estimates of variance components and genetic parameters for average somatic cell score according to the univariate model.

	Model 1. Test Day Average	Model 2. Average Total Lactations
Genetic effect variance	0.2577 (0.013)	0.4678 (0.011)
Permanent effect variance	0.2522 (0.010)	0.3511 (0.017)
Residual variance	0.5389 (0.005)	1.4785 (0.004)
Heritability	0.246 (0.011)	0.204 (0.005)
Repeatability	0.486 (0.005)	0.356 (0.003)
Number of records	57,297	340,654

(Standard error in brackets).

**Table 3:** Estimates of variance components, genetic parameters, and principal components of the genetic matrix for the average somatic cell score according to the multiple models.

	Model 3. Average Each Parity			Model 4. Test Day Average		
Genetic Var First parity	0.257 (0.016)			0.696 (0.020)		
Genetic Var Second parity	0.399 (0.019)			0.863 (0.022)		
Genetic Var Third parity	0.382 (0.019)			0.813 (0.023)		
Permanent Var	0.255 (0.010)			0.308 (0.011)		
Residual Var First parity	0.536 (0.011)			1.450 (0.006)		
Residual Var Second parity	0.435 (0.011)			1.217 (0.006)		
Residual Var Third parity	0.412 (0.012)			1.160 (0.007)		
Heritability First parity	0.245 (0.013)			0.284 (0.007)		
Heritability Second parity	0.366 (0.015)			0.361 (0.007)		
Heritability Third parity	0.365 (0.016)			0.356 (0.008)		
Repeatability First parity	0.488 (0.011)			0.409 (0.004)		
Repeatability Second parity	0.601 (0.010)			0.491 (0.004)		
Repeatability Third parity	0.607 (0.012)			0.491 (0.005)		
Rg first and second parity	0.767 (0.022)			0.456 (0.015)		
Rg first and third parity	0.538 (0.034)			0.178 (0.021)		
Rg second and third parity	0.854 (0.011)			0.658(0.011)		
Principal Components Analysis Genetic variance explained %	PC1	PC2	PC3	PC1	PC2	PC3
	86.5	13.4	0.01	64.8	25.6	9.6
Eigenvector 1	0.417	−0.846	0.331	0.389	−0.843	0.371
Eigenvector 2	0.666	0.038	−0.745	0.697	0.006	−0.717
Eigenvector 3	0.618	0.531	0.580	0.603	0.538	0.590

(Standard error in parentheses).

**Table 4:** Estimates of the correlations between the breeding values of the goats (EBV) for somatic cell counts in different parities (expressed as the average of lactation or each test day).

EBV SCC Average of Each Lactation			EBV SCC on Each Test Day		
First Parity	Second Parity	Third Parity	First Parity	Second Parity	Third Parity
1.000	0.870	0.730	0.757	0.662	0.485
	1.000	0.972	0.554	0.769	0.697
		1.000	0.407	0.752	0.735
			1.000	0.622	0.389
				1.000	0.813
					1.000

**Table 5:** Results of principal component analysis, the conformation of index (Ipc) and correlations between Ipc and original breeding values for each parity.

Principal Component	Average for Each Parity			Test Day Average		
	PC1	PC2	PC3	PC1	PC2	PC3
Eigenvalues $\lambda_{i}$	2.719	0.281		2.232	0.630	0.138
% Explained variance	90.6	9.4		74.4	21.0	4.6
Eigenvector ev parity 1	0.551	0.790		0.505	0.816	−0.281
Eigenvector ev parity 2	0.604	−0.156		0.638	−0.134	0.758
Eigenvector ev parity 3	0.576	−0.592		0.581	−0.562	−0.588
Ipc1	0.551 × EBV_1_ + 0.604 × EBV_2_			0.505 × EBV_1_ + 0.638 × EBV_2_		
Ipc2	0.790 × EBV_1_ − 0.156 × EBV_2_			0.816 × EBV_1_ − 0.134 × EBV_2_		
IpcT	Ipc1 + Ipc2			Ipc1 + Ipc2		
Correlation between EBVi and Ipc	Ipc1	Ipc2	Ipct	Ipc1	Ipc2	Ipct
First parity	0.901	0.419	0.947	0.754	0.648	0.868
Second parity	0.995	−0.123	0.982	0.953	−0.106	0.915
Third parity	0.247	−0.530	0.910	0.868	−0.446	0.761

## Data Availability

The data sets employed in this study are the property of the ACRIFLOR (Asociación Nacional de Criadores de Ganado Caprino de Raza Florida-National Association of Florida Goat Breeders) and were provided for scientific purposes under a specific collaboration arrangement. The data set could be provided for scientific purposes to further authors under reasonable request in the ACRIFLOR technical department.
